# InFoRM: a unified inverse and forward model for sensorimotor control

**DOI:** 10.1038/s41598-026-39944-z

**Published:** 2026-03-09

**Authors:** Myriam Lauren de Graaf, Lena Kloock, André Schwarze, Meike Gerlach, Andrea Arensmann, Kim Joris Boström, Ricarda I. Schubotz, Heiko Wagner

**Affiliations:** 1 Department of Movement Science, University of Münster, Horstmarer Landweg 62b, 48149 Münster, Germany; 2 Otto Creutzfeldt Centre for Cognitive and Behavioural Neuroscience, University of Münster, Fliednerstraße 21, 48149 Münster, Germany; 3 Centre for Data Science and Complexity (CDSC), University of Münster, Corrensstraße 2, 48149 Münster, Germany; 4 Institute of Psychology, University of Münster, Fliednerstraße 21, 48149 Münster, Germany

**Keywords:** Motor control, Internal models, Forward model, Inverse model, Reservoir computing, Neural networks, Engineering, Neuroscience

## Abstract

Sensorimotor control models traditionally consist of two types of internal models: *inverse* models, which compute the motor commands needed to reach a desired movement goal, and *forward* models, which predict the resulting sensory feedback. These models are usually considered separate entities, but it is unclear whether such separation exists in the nervous system. Additionally, maintaining separate networks may be more computationally expensive. Therefore, we investigated whether these functions could be executed within a single neural circuit: an *inverse-forward-recognition model* (InFoRM). We implemented InFoRM using neural networks and compared their ability to reproduce cyclic reaching movements with that of control architectures based on classical, separated inverse and forward models. Desired movement trajectories were represented by recorded three-dimensional kinematics, while efferent (muscle activation) and afferent (muscle length and velocity) signals were obtained through inverse dynamics. Our findings show that InFoRM significantly outperforms control architectures across various conditions, while requiring fewer resources. The network is also able to morph to untrained movement directions, generating motor commands and predicted feedback that had not been learned. These findings demonstrate the computational advantages of integrating inverse and forward processes within a single neural network, suggesting that such unified sensorimotor models may be worthwhile to explore further.

## Introduction

Sensorimotor models have long provided a foundational framework for understanding how the nervous system generates coordinated movements. These models describe the transformation from a desired motor outcome to the appropriate muscle commands, as well as the ongoing evaluation of movement accuracy through the comparison of predicted and actual sensory feedback. Central to most contemporary sensorimotor control theories is the concept of *internal models*: hypothetical functional centres in the central nervous system that mimic the relation between the input and output of the motor system^1–3^. By conceptualising these transformations, internal models enable researchers to develop and test hypotheses about the neural and computational mechanisms underlying movement generation.

**Fig. 1:**
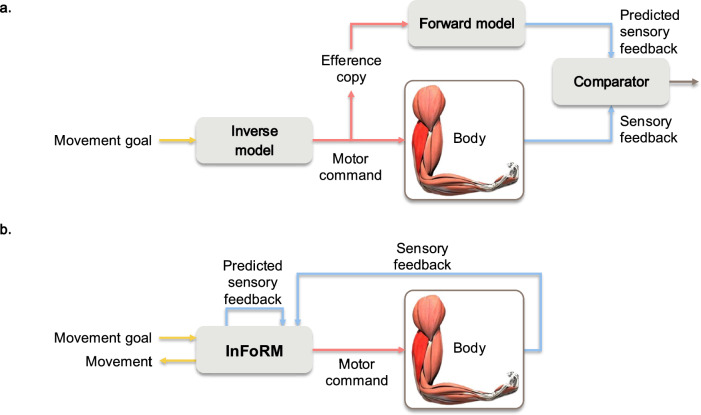
Comparison of (**a**) the classic sensorimotor control model and (**b**) the investigated inverse-forward-recognition model (InFoRM).

Classic sensorimotor models^1,3–5^^, etc.^ (shown in Fig. 1a) generally employ two types of internal models: inverse and forward models. The *inverse model*, also known as the *controller*, transforms the desired motor output (the ‘movement goal’) to the necessary motor commands that are to be sent to the muscles, whereas the *forward model* predicts the corresponding sensory feedback based on the generated motor commands. These components are theorised to operate in tandem, in a hierarchical construction, with efference copies of motor signals feeding into the forward model to refine predictions and correct errors^1,6,7^. Internal models are widely employed in influential theoretical frameworks, including predictive coding^8,9^, the predictive brain framework^4^, common-coding^10^, and the theory of event coding^11^, thereby underpinning much of our current understanding of how the brain plans and predicts actions.

While inverse and forward models have traditionally been treated as separate systems in sensorimotor models, direct evidence for this separation is lacking. Several regions have been considered as potential locations for inverse and forward models, but none of them have been directly identified (see discussion). Among these regions, the cerebellum and parietal cortex have been identified as candidate regions for both inverse and forward models^12–14^, leaving open the possibility that a single neural circuit could implement both functions. Additionally, it is often suggested that inverse and forward models are tightly linked and that they work with a high degree of coordination^7,15–17^. This raises the question of whether forward and inverse models are necessarily distinct entities, or if they could rather be emergent features of a single, unified circuit.

A potential benefit of unified models is that they may be more efficient, as maintaining separate neural circuits for inverse and forward computations could impose excessive metabolic and computational costs. Studies involving multi-task and multi-output learning indicate that training multiple signals simultaneously within a single network can reduce inference time and improve performance^18–22^. By learning multiple tasks together, networks can exploit shared representations between correlated tasks, and uncorrelated tasks may further improve generalisation by acting as a source of beneficial noise^19^. Separate networks cannot exploit these advantages and consequently require a larger number of neurons and synaptic connections, increasing their metabolic costs^23,24^. These findings suggest that implementing inverse and forward computations within a single neural circuit may be more efficient than two separate circuits.

Taken together, these considerations motivate the exploration of unified models as an alternative architectural implementation of sensorimotor control. To explore this idea, we designed a model that serves both as a controller for muscular activity (the inverse function) and as a predictor of the corresponding sensory feedback (the forward function). In addition, we posit that the model could infer the intended sensorimotor goal from the available internal signals, i.e. that it can reconstruct the desired movement input (the recognition function). We thus designed the inverse-forward-recognition model (InFoRM), depicted in Fig. 1b. The InFoRM model is expected to be able to *morph* between learned behaviours, i.e. to generalise from trained to untrained movements. This would allow the sensorimotor system to perform movements that it has never learned before and to simultaneously predict corresponding sensory feedback that it has never experienced before.

To investigate the feasibility of this novel sensorimotor control model, we adopt artificial neural networks, specifically reservoir computing, as a practical modelling framework. Reservoir computers are a type of recurrent neural network that preserve the rich, recurrent connectivity characteristic of biological neural circuits, while avoiding the computational challenges associated with backpropagation by optimising only the final readout layer^25–27^. We have previously shown that reservoir computers can learn to predict both efferent and afferent data, including EMG signals, muscle activation, and arm angles^28,29^. InFoRM’s expected morphing capabilities have also been demonstrated in reservoir computing networks^28–30^. Reservoir computing, therefore, offers a suitable framework for implementing and evaluating the proposed model.

In this study, we investigate the feasibility of the inverse-forward-recognition model (InFoRM), which captures both the predictive and generative aspects of sensorimotor control within a single architecture. Using real-life data from cyclic reaching movements, we evaluate whether InFoRM can simultaneously reproduce the motor command (efference), the predicted sensory consequences (afference), and the intended movement goal. To this end, we implement InFoRM within a reservoir computing framework and benchmark it against various control architectures that model inverse and forward computations as separate, hierarchical units, in line with traditional sensorimotor control theories.

## Methods

To provide suitable training data for our InFoRM and Control networks, we recorded kinematics of controlled, cyclic reaching movements and derived the necessary signals using a musculoskeletal model. The following sections detail the experimental protocol, data acquisition procedures, and modelling approach.

### Data acquisition

Twelve healthy volunteers (7 female, 5 male; age: $22.8\pm 3.5$ years; height: $175.5\pm 12.2$ cm; weight: $72.3\pm 12.0$ kg; 1 left-handed) took part in the experiment. All participants provided written informed consent prior to the start of the measurements. The experimental procedures were approved by the Ethics Committee of the Faculty of Psychology and Sports Science at the University of Münster (#2024-19-MG), and performed in accordance with their regulations.

**Fig. 2:**
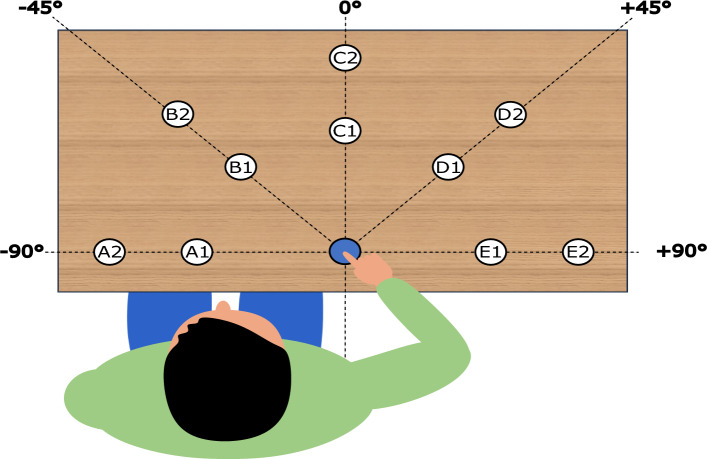
Experimental set-up for a right-handed participant. The start position is indicated in blue. The targets, indicated in white, were placed in five directions (labelled A through E for $-90$°, $-45$°, $0$°, $+45$°, and $+90$°), at either 25 or 40 cm from the start target (labelled 1 and 2, resp.).

Eleven targets (ø2.5 cm) were arranged on a tabletop with a horizontal surface: one central target served as the start position, and the remaining ten targets were positioned along five directions ($-90$°, $-45$°, $0$°, $+45$°, and $+90$°, where $0$° is straight ahead) and at two distances (25 and 40 cm) from the central target (see Fig. 2). The targets were labelled with letters A to E according to their directions (from A for $-90$° to E $+90$°) and a number 1 or 2 indicating the closest and furthest target, respectively. All targets were visibly marked on the tabletop, and their positions remained fixed throughout the experiment. Participants were seated at the table, with the shoulder of their dominant arm aligned with the central start target. They were instructed to adjust the distance between the chair and the table so that they could comfortably reach all targets, while keeping their back in contact with the backrest throughout the task.

Participants performed continuous back-and-forth reaching movements between the central target and one of the peripheral targets, using their dominant hand. Movements were paced by a metronome set to either 90 or 120 beats per minute, and participants were instructed to tap each target on the beat (i.e. full movement cycles were performed at 1.0 and 0.75 Hz, respectively). This resulted in 20 unique conditions (5 directions $\times$ 2 amplitudes $\times$ 2 frequencies) that were performed in randomised order. To supplement the test data with trials with natural transitions between the targets, each subject performed extra conditions where all targets were tapped in a self-decided random order at least once, with either one or three consecutive taps per target. This was done for both frequencies, for a total of 4 extra trials.

Kinematic data were recorded using an Xsens Link suit (Movella Inc., Henderson, NV, USA), equipped with 17 inertial measurement units (model MTx, size 36 $\times$ 24.5 $\times$ 10 mm, mass 10 g) at a sampling rate of 240 Hz. Recordings began once the participants had visibly synchronised their movement with the metronome. Twelve movement cycles were recorded to guarantee the availability of ten complete cycles per trial, providing enough training data, while preventing fatigue-related effects.

### Data processing

The recorded kinematics were used as input for the 3D musculoskeletal model Myonardo®^31^ embedded in Computed MyoGraphy (CMG, version 6.4.0, Predimo GmbH, Münster, Germany). CMG has previously been validated^31,32^ and used^29,32–36^. More details, including the filtering performed within the musculoskeletal model, can be found in Supplementary Section A. Using inverse dynamics and static optimisation, the model calculated the muscle activation and sensory feedback that could reproduce the recorded movements, providing the afferent and efferent signals used to train the networks. The model returned outputs downsampled to 120 Hz.

The efferent signal consisted of the calculated muscle activations, which are theoretically bound between 0 and 1. To account for inter-individual differences in movement strategies, the eight muscles with the largest activation range were chosen separately for each individual. This provided a reduced yet sufficiently rich representation of the muscle activation space, capturing the dominant task-related activity while avoiding inclusion of muscles with minimal involvement. An overview of the muscles included for each subject can be found in Supplementary Table S1. For the afferent signals, we used the calculated muscle length and muscle velocity for each of the included muscles.

For the movement goal, the 3D coordinates of the finger marker were taken as a proxy for the endpoint effector. This equates to the first level of computational problems that the CNS needs to solve, as described by Kawato et al.^37^, whereas the efferent signal is the result of the third and final computational step. Drift was removed from these kinematics signals, and the start target position was moved to the origin to prevent unnatural jumps in the goal data.

The resulting signal set thus comprised 3 goal signals, 8 efferent signals, and 16 afferent signals. All signals in the main trials were cut to include 10 complete cycles, with a cycle starting and ending at the central target. For the extra trials with natural transitions, all complete cycles were included.

#### Data partitioning

The full dataset thus consisted of 20 main trials with 10 oscillations per trial, plus 4 extra test trials. The data were partitioned into train, validation and three different test sets. The training and validation data consisted of oscillations 1–6 and 7–8, respectively, of the trials in directions A, C, and E. The *Basic* test set consisted of oscillations 9–10 of these three cardinal directions. The *Morphing* test set contained all oscillations of the movements in directions B and D. Finally, the *Natural Transitions* test set was made up of the four extra trials where subjects went to each target in a self-chosen order.

The data in the Basic and Morphing sets were merged in a randomised order, with each condition represented once, to form a single, large time series per signal. For each of the test signals, one random trial from within the set was added to the start of the signal, serving as the initialisation period for the network. This first section was excluded when calculating the model performance. The trials in the Natural Transitions were not merged, but, as in the other test signals, one of the 20 standard conditions was added at the start so that the network’s transient period could be excluded from the evaluation. Each signal was individually normalised using its minimum and maximum values from the training dataset, and re-normalised for final error calculation and data visualisation.

### Neural network models

To contextualize the performance of the proposed InFoRM network, we compared it with several widely used baseline sensorimotor control models. For the neural network implementation of these models, we employed echo state networks (ESNs)^26^, a type of recurrent neural network characterised by a fixed, randomly connected reservoir and a trainable linear output layer. Specifically, we used ESNs with a recursive least-squares training algorithm^38^, based on the principle of FORCE learning^39^. The network architectures are outlined below, with detailed descriptions and network equations provided in Supplementary Section B.1. A comparative overview of all network variants, including their architectural characteristics and evaluation metrics, is provided in Supplementary Table S2.

**Fig. 3:**
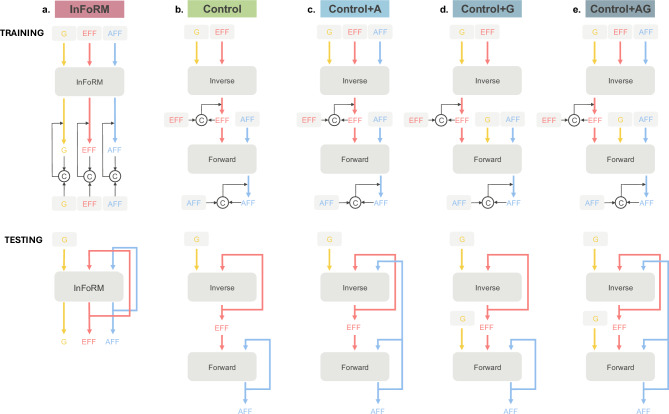
Network schematics during training (top) and testing (bottom) for (**a**) the InFoRM network, (**b**) the baseline Control network, (**c**) Control+A, the control network with additional afference supplied to the inverse network, (**d**) Control+G, the control network with additional goal information supplied to the forward network, and (**e**) Control+AG, the control network with additional afference to the inverse network and goal information supplied to the forward network. Large grey boxes denote the three neural network circuits: InFoRM, Inverse, and Forward. The yellow lines indicate the goal information (‘G’) represented by the desired movement kinematics, the red lines depict the efference (‘Eff’), represented by the muscle activation, and the blue lines depict the afference (‘Aff’), represented by the muscle length and velocity. Signals shown inside light grey boxes correspond to target signals, whereas unboxed signals represent network outputs. Circles labelled C denote comparator units that compute the difference between the two incoming signals. The resulting error signal is used to update the synaptic weights, as indicated by the thin black arrows.

The InFoRM network (see Fig. 3a) consists of a single reservoir that outputs three types of signals: the desired kinematics (i.e. the movement goal, depicted in yellow), the required muscle activation (efference, depicted in red), and the expected resulting sensory feedback (afference, depicted in blue). All three signals can either be fed back into the network as output feedback (as, e.g., the efferent and afferent signal during testing (bottom) in Fig. 3a) or this output feedback can be overwritten to provide an external input (as, e.g., all signals during training (top) in Fig. 3a, and the goal signal during testing (bottom) in Fig. 3a). To provide accurate feedback from the outset, all signals were overwritten during the training process. During testing, we only used the situation depicted in Fig. 3 (i.e. with the goal as external input, and the efference and afference as output feedback), as it most closely resembles the natural situation. However, any combination of output feedback and external input is possible for the InFoRM network.

The baseline Control network (see Fig. 3b) is structured like the classic sensorimotor control models, consisting of separate inverse and forward networks. The inverse network maps the movement goal to the muscle activation (efference), and the forward network maps this muscle activation to the sensory feedback (afference). Both networks receive output feedback. To align with the InFoRM network’s training configuration, output feedback signals were replaced by target signals during training (see Fig. 3b, top).

To test whether performance differences between the InFoRM and Control network stemmed from their respective architectures and not their access to input information, we designed and evaluated three intermediate control variants that gradually bridge the gap between the Control and InFoRM architectures. Specifically, these variants introduce key features of the InFoRM network into the Control network in a stepwise fashion: (1) Control+A, in which the inverse network also receives afferent feedback (see Fig. 3c); (2) Control+G, where the forward network is provided with goal information as input (see Fig. 3d); and (3) Control+AG, which combines both modifications (see Fig. 3e).

To represent movement planning, all networks received not only the goal information of the current time point but also of the upcoming 300 ms via a look-ahead window (corresponding to 30%, respectively 40% of a complete movement cycle), matching the planning horizons observed in untrained individuals performing continuous tracking tasks^40^.

### Network training

All networks were trained using a recursive least squares algorithm^38^. During the training process, the output feedback was overwritten by the target output signals to provide correct output feedback from the beginning (see Fig. 3, top). Noise was added to these target signals to improve robustness to small output fluctuations. During the training process, hyperparameters were optimised via Bayesian optimisation (see Supplementary Table S3 for an overview). These included the number of training iterations, the number of neurons in each network, connection strength, learning rate, noise scaling, and more. Additional details on the training process and the hyperparameter optimisation can be found in Supplementary Section B.2.

### Model evaluation

Final model performance was assessed using the three aforementioned test sets. The Basic test set consists of the unseen oscillations of the trained directions (A, C, E) and tested generalisation within learned movements. The Morphing test set contained the held-out directions (B and D) and was intended to test the morphing capabilities of the network, i.e. generalisation to untrained directions. Finally, the Natural Transitions set contains unseen oscillations in all directions, and thus evaluated both the prediction and morphing capabilities of the network as well as whether it can generalise to natural transitions between conditions (vs. the cut-and-pasted signals in the other test sets). The performance of the networks was evaluated using the relative root mean squared error (rRMSE), normalised by the range of the target signal, and Pearson’s correlation coefficient. These metrics were chosen to capture both the magnitude of the prediction errors as well as the alignment between the predicted and true signals. To ensure robust and reproducible results, each model was trained and evaluated 10 times across independent instances, using the same set of random seeds for every subject and network type (see Supplementary Section B.2 for the seed numbers), with final performance metrics reported as the average across all seeds.

### Statistical analysis

Statistical analysis was performed in MATLAB (version 2024a, The MathWorks, Inc., Natick, Massachusetts, United States) using the $\texttt {kbstat}$ toolbox (version 1.1.4)^41^. We performed a generalised linear mixed model (GLMM) analysis instead of a more traditional repeated-measures analysis of variance (rANOVA) to statistically evaluate the data in an optimal and consistent manner, despite non-normal data distributions. GLMM fits were performed individually for the performance measures (rRMSE/correlation) for each of the three test sets (Basic/Morphing/Natural Transitions), as well as for each of the two hyperparameters relevant for the computational efficiency (total network size $N$/number of training iterations $n_\text {iter}$). This resulted in a total of $3 \times 2 +2 =8$ fits.

As the hyperparameters were optimised separately for each combination of subject identity and network type, we tested the dependency of these dependent variables on the independent variable $\texttt {Network}$, conditioned on the random variable $\texttt {Subject}$. In Wilkinson notation, the statistical model with the fewest variables that yielded the best fit according to the Akaike (AIC) and Bayesian (BIC) information criteria was

1 $$\begin{aligned} \texttt {Y} \sim \texttt {Network} + (\texttt {Network}|\texttt {Subject}), \end{aligned}$$

where $\texttt {Y}$ represents either of the two relevant hyperparameters, and where we used a Gamma distribution with a log link function in both cases, as these variables are bounded from below by zero and their distribution is skewed.

We then tested for the dependency of a given performance measure (correlation or rRMSE), which thus became our dependent variable, on the network type $\texttt {Network}$, which became our independent variable, conditioned on the subject identity $\texttt {Subject}$ and network instance $\texttt {Instance}$, which became our two random variables. However, neither including $\texttt {Instance}$ as a random effect, nor adding a random slope for $\texttt {Subject}$, nor including both improved the model fit in terms of AIC and BIC. The least complex, best-fitting model was

2 $$\begin{aligned} \texttt {Y} \sim \texttt {Network} + (\texttt {1}|\texttt {Subject}), \end{aligned}$$

where $\texttt {Y}$ was either rRMSE or correlation for each of the three test sets. In the case of the rRMSE, we used a Gamma distribution with a log link function, as this variable is non-negative and its distribution is skewed. For the correlation, the values are bounded within the interval $[-1, 1]$ and their probability distribution is even more skewed. We therefore first applied a Fisher transform (inverse hyperbolic tangent) and then performed a GLMM fit using a normal distribution and an identity link function.

To prevent potential distortion of the GLMM fits, extreme statistical outliers were removed before each fit by excluding any point outside 1.5 times the $\left( 0.25, 0.75\right)$ interquartile range. This resulted in the exclusion of $1.4\%$ of the correlations, $7.1\%$ of the rRMSEs, and $0.8\%$ of the hyperparameters. The GLMM fits were performed using the $\texttt {kbstat}$ toolbox, which employs MATLAB’s $\texttt {fitglme}$ function, with the maximum pseudo-likelihood (MPL) method and effects coding. The quality of each GLMM fit was verified using diagnostic plots, paying particular attention to the normality of the residual distribution and homoscedasticity. Post hoc pairwise comparisons were also performed using the $\texttt {kbstat}$ toolbox and statistically corrected using the Holm-Bonferroni method. The global significance level was set to $\alpha = 0.05$.

As is common in GLMM analyses, the reported values for the estimates of the dependent variables under the various conditions are the estimated marginal means (EMMs) and corresponding 95% confidence intervals resulting from the respective GLMM fit. Effect sizes for both the main effects and post hoc comparisons were calculated as partial eta squared ($\eta _p^2$) and interpreted via Cohen’s rule of thumb, where [0.01, 0.06, 0.14] are the lower limits that signify small, medium and large effect sizes, respectively^42^. Partial eta squares can be interpreted as the proportion of the total variance explained by the variable in question once the effects of the other variables have been removed.

## Results

### Accuracy

**Fig. 4:**
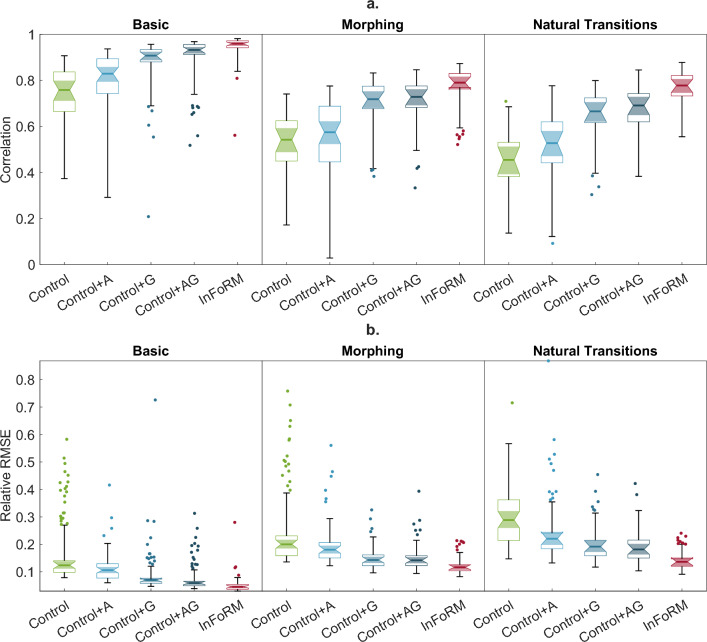
Performance of the investigated networks on the three test sets. (**a**) Correlations and (**b**) rRMSE are depicted for each network for the Basic (left), Morphing (centre) and Natural Transitions (right) test sets. The centre lines of the box charts indicate the estimated marginal means, with the coloured notch depicting the 95% confidence interval around it. The full box indicates the 25% and 75% interquartile ranges, and the whiskers the outlier-free data range. The coloured markers depict the outliers removed by the generalised linear mixed model. Significant differences between the networks are reported in Supplementary Table S4, with a condensed version in Table 2.

Across all three test sets and both outcome measures, the InFoRM architecture consistently outperformed all control networks, achieving higher correlations and lower rRMSE values (see Fig. 4) and Supplementary Table S2. The GLMMs revealed a significant main effect of network architecture on performance for both metrics across all three test sets (all $p< 0.001$), with effect sizes ranging from large to very large (see Table 1). Post hoc comparisons showed that InFoRM significantly outperformed both the standard Control network and all augmented Control+ variants (see Table 2). All other post hoc comparisons were also significant at the $\alpha =.05$ level, except for the comparison between Control+G and Control+AG on the Morphing test set (see Supplementary Table S4). On the Basic test set, all networks achieved correlations of $> 0.75$ and rRMSEs of $< 0.12$, with InfoRM achieving both the highest correlation (EMM $[95\% \, \text {CI}] = 0.96 [0.95 \, 0.97]$) and the lowest rRMSE ($0.05[0.04 \, 0.05]$).

**Table 1:** ANOVA results for the main effect of network architecture on rRMSE and correlation across the three test sets.

Measure				
Test set	Outcome	${F}$(df)	${p}$	Effect size
Basic	rRMSE	349.83 (4, 537)	$<0.001$	Very large
	correlation	354.77 (4, 580)	$<0.001$	Very large
Morphing	rRMSE	176.21 (4, 556)	$<0.001$	Large
	correlation	281.36 (4, 583)	$<0.001$	Very large
Natural Transitions	rRMSE	224.50 (4, 566)	$<0.001$	Very large
	correlation	485.52 (4, 590)	$<0.001$	Very large

**Table 2:** Key post hoc comparisons examining the effect of network architecture on rRMSE and correlation. This table reports only the comparisons between InFoRM and the standard Control network, and between InFoRM and the best-performing Control+ network, which was Control+AG for all six cases. Full comparisons can be found in Supplementary Table S4.

Measure		Comparison							
Test set	Outcome	DF1	DF2	InFoRM v. Control			InFoRM v. Best Control+		
				*F*	*p*	$\eta _p^2$	*F*	*p*	$\eta _p^2$
Basic	rRMSE	1	537	1005.8	$<0.001$	0.65	72.313	$<0.001$	0.12
	correlation	1	580	1112.2	$<0.001$	0.66	82.021	$<0.001$	0.12
Morphing	rRMSE	1	556	551.94	$<0.001$	0.50	77.669	$<0.001$	0.12
	correlation	1	583	788.32	$<0.001$	0.57	77.502	$<0.001$	0.12
Natural Transitions	rRMSE	1	566	836.47	$<0.001$	0.60	120.97	$<0.001$	0.18
	correlation	1	590	1538	$<0.001$	0.72	184.35	$<0.001$	0.24

### Generalisation and morphing

**Fig. 5:**
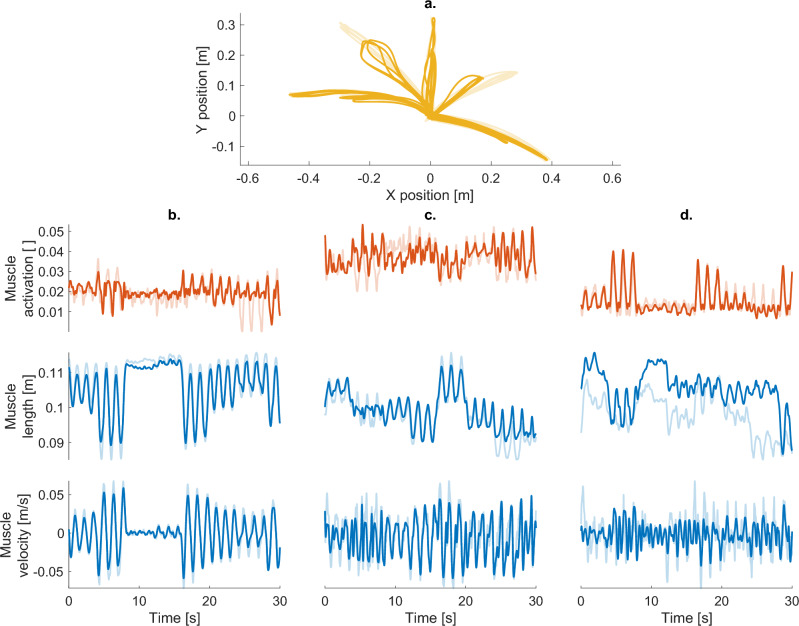
Example outputs (opaque) and targets (transparent) for the InFoRM network for a random subject, with (**a**) the movement goal and (**b**–**d**) the muscle activation (top), muscle length (middle) and muscle velocity (bottom) shown for (**b**) the best (correlation $=0.94$ rRMSE $= 0.07$), (**c**) the median (correlation $=0.78$ rRMSE $= 0.11$) and (**d**) the worst (correlation $=0.76$ rRMSE $= 0.18$) predicted muscle.

All networks were also able to generalise beyond the trained directions. On the Morphing test set, networks successfully interpolated between the trained target directions, reproducing movements in untrained directions with reduced but still relatively high accuracy (see Fig. 4, central panels). The InFoRM network achieved the highest correlations (EMM $[95\% \, \text {CI} ] = 0.79 [0.76 \, 0.82 ]$) and lowest rRMSE ($0.20 [0.19 \ 0.22 ]$) of all investigated architectures, with the best-performing control network (Control+AG) scoring $0.73 [0.69 \, 0.76 ]$ and $0.14 [0.13 \, 0.15 ]$, respectively. On the Natural Transitions test set, the performance decreased slightly compared to the Morphing task (see Fig. 4, right-hand panels). InFoRM retained a clear advantage over the control models, on both the correlation ($0.78 [0.75 \, 0.81 ]$ vs. $0.69 [0.65 \, 0.73 ]$ for Control+AG) and rRMSE ($0.14 [0.12 \, 0.15 ]$ vs. $0.18 [0.16 \, 0.20 ]$ for Control+AG). An example network output of the InFoRM network on this third test set is shown in Fig. 5. Overall, these results demonstrate that InFoRM generalises better to increasingly complex and realistic test conditions.

### Hyperparameters

**Fig. 6:**
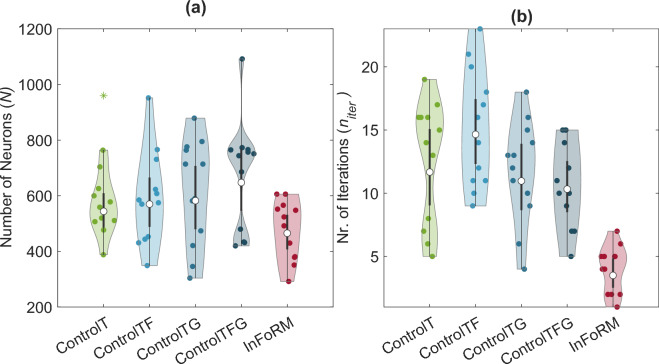
Optimised values for the two hyperparameters that impact the efficiency of the networks: (**a**) total neuron number, and (**b**) number of training iterations. Each marker represents the optimised hyperparameters for one subject, based on an average over five iterations. Statistical outliers are denoted by asterisks. Significant differences between the networks are reported in Supplementary Table S5. For the control networks, the total neuron number is the sum of the inverse and forward networks. The neuron numbers are depicted per subnetwork in Supplementary Fig. S1.

The final hyperparameter values differed between the network architectures, as each network was optimised individually. We focus on the total neuron count and the number of training iterations as these most directly impact network efficiency (see Supplementary Fig. S1 for an overview of all hyperparameters). The optimised InFoRM networks contained fewer total neurons (EMM $[95\% \, \text {CI} ] = 465.47 [407.44 \, 531.77]$) than the Control network ($544.15 \, [486.08 \, 609.16]$) and all Control+ variants (see Fig. 6a), although none of these differences reached statistical significance (all $p>0.05$, see Supplementary Table S5). InFoRM needed significantly fewer training iterations ($3.5 [2.53 \, 4.85 ]$) compared to the control network ($11.68 [9.04 \, 15.08 ]$, $F(1,55)=45.517$, $p< 0.001$) and all Control+Networks (see Fig. 6b and Supplementary Table S5).

## Discussion

In this study, we investigated whether sensorimotor control models, which are traditionally thought to consist of topologically distinct inverse and forward components, can be implemented as a single unified network: the InFoRM network. We addressed this question by evaluating the performance of an InFoRM-based neural network on a cyclic reaching task and comparing it to a series of control architectures that consisted of separate inverse and forward networks. The InFoRM network performed significantly better than the classic hierarchical architectures on all three test sets, showing higher correlations and lower relative root mean square errors compared to the standard Control network and all Control+ variants. These advantages were maintained even under challenging generalisation conditions, including interpolation to unseen movement directions (Morphing test set) and naturalistic transitions between targets (Natural Transitions test set). Crucially, InFoRM significantly outperformed even the Control+AG network, which received identical external information (goal, efferent, and afferent signals), indicating that InFoRM’s superior performance cannot be attributed solely to its access to more information. InFoRM also demonstrated greater computational efficiency, requiring a significantly smaller number of training iterations and a non-significantly lower number of neurons. Our findings thus show that integrating both functions offers a computational advantage over the classic hierarchical network architectures.

These theoretical advantages of a unified inverse–forward architecture raise the question of whether such a model could plausibly be implemented within the central nervous system in light of current neuroanatomical evidence. Neurophysiological, lesion, and stimulation studies have associated inverse and forward model functions with different neural regions, a finding often taken to imply spatially distinct internal models. The forward-model-related functions are primarily linked to the cerebellum^12,13,43–45^ and the parietal cortex^12,46^, while inverse-model-related functions have been associated with a broader set of regions, including motor, premotor, parietal, basal ganglia, and cerebellar circuits^1,13,16,46,47^. However, such localisation is frequently based on contrasting brain activity across tasks thought to differentially engage inverse or forward control, a methodology that is inherently unable to detect unified inverse–forward models, which are expected to be active during both task conditions. Several regions show engagement during both feedforward and feedback control^14^, making them plausible candidates for unified inverse–forward motor control. The regions that were identified as selectively active may instead reflect downstream processing of either the inverse or forward outputs of the unified model rather than the site of the internal/forward model itself.

Other insights come from lesion and stimulation studies, which have identified the cerebellum as a key contributor to only predictive control, which is often taken as evidence that it implements a forward model^6,48^. However, these effects could also arise from disruptions in proprioception or sensorimotor integration, processes in which the cerebellum is known to play an important role^49,50^. Taken together, existing evidence implicates multiple neural circuits for both inverse and forward functions, but the available evidence does not rule out the existence of a unified InFoRM-like neural circuit.

Because InFoRM does not explicitly implement separate forward and inverse computations, but instead has them emerge from a single recurrent circuit, it embodies a generative scheme close to predictive-processing formulations. In predictive processing, active inference applies the same principle to motor control: the generative model remains forward, predicting the sensory consequences of intended movements, while the role of an inverse model is realised implicitly through descending proprioceptive predictions that are fulfilled via reflex arcs^51^. Unlike hierarchical predictive-coding models that exchange predictions and errors across multiple levels, InFoRM’s reservoir captures bidirectional relationships between sensory and motor states within a single recurrent population, offering computational simplicity and fast convergence. Classic hierarchical architectures, in turn, may better capture biological constraints and context-dependent modulation more faithfully. This suggests that hierarchical and unified approaches address complementary aspects of sensorimotor prediction and control.

While the present results support the feasibility and potential benefits of a unified model, several caveats must be acknowledged. First, the present comparison was conducted within a reservoir computing framework, in which internal recurrent weights remain fixed after random initialisation. InFoRM receives output feedback from both efferent and afferent signals, which may indirectly modulate effective connectivity more strongly than in the control subnetworks, that receive output feedback from fewer signals. However, InFoRM also outperformed the Control+AG architecture, which also receives both signal types. Whether similar advantages persist in recurrent networks with fully trainable internal weights remains an open question. Second, the reservoir computing approach, while powerful, abstracts away many biological details such as synaptic plasticity mechanisms, neuromodulation, and heterogeneous neuron types. Furthermore, no delays have been modelled. Our architecture assumes clear, full, and synchronous access to both efferent and afferent information, while biological systems often work with incomplete and delayed signals. Future work could explore how these factors influence the capacity for integrated forward-inverse modelling in more biologically realistic networks.

This study demonstrates that forward and inverse sensorimotor computations can be effectively integrated within a single neural network architecture. The InFoRM network consistently outperformed the classic hierarchical networks across multiple test sets, including conditions requiring generalisation to untrained movement directions, despite using fewer training iterations. Our results suggest that InFoRM’s advantage arises not merely from access to information, but from the integrated processing and recurrent dynamics within a unified circuit. Overall, these findings support the feasibility and computational benefits of unified sensorimotor architectures, highlighting a complementary perspective to hierarchical predictive-coding models and opening avenues for future investigations.

## Supplementary Information

Supplementary Information.

## Data Availability

The data underlying this study are available from the corresponding author upon request.
