# Supplementary material for: InFoRM: a unified inverse and forward model for sensorimotor control
Source: Sci Rep. 2026 Mar 9;16:8490. doi: 10.1038/s41598-026-39944-z (PMC12972123; doi:10.1038/s41598-026-39944-z)
Supplement: Supplementary file 1 — Supplementary Information. [file 41598_2026_39944_MOESM1_ESM.pdf]

InFoRM: A Unified Inverse and Forward Model for Sensorimotor Control

Myriam Lauren de Graaf[1,2,3*], Lena Kloock[1], André Schwarze[1], Meike Gerlach[1], Andrea Arensmann[1], Kim Joris Boström[1], Ricarda I. Schubotz[2,4], and Heiko Wagner[1,2,3]

[1]Dept. of Movement Science, University of Münster, Horstmarer Landweg 62b, 48149 Münster, Germany
[2]Otto Creutzfeldt Centre for Cognitive and Behavioural Neuroscience, Fliednerstraße 21, 48149, Münster, University of Münster, Germany
[3]Centre for Data Science and Complexity (CDSC), University of Münster, Corrensstraße 2, 48149, Münster, Germany
[4]Institute of Psychology, University of Münster, Fliednerstraße 21, 48149 Münster, Germany
[*]Corresponding author: mdegraaf@uni-muenster.de

A Musculoskeletal Model

We used the 3D musculoskeletal model Myonardo®[1] embedded in Computed MyoGraphy (CMG, version 6.4.0, Predimo GmbH, Münster, Germany) that has previously been validated[1,2] and used[2–7].

The musculoskeletal model consists of 36 segments, 38 joints (with degrees of freedom varying between 0 and 6), and 692 Hill-type muscles. The relative mass and size of the segments, and the muscular origins and insertions were determined based on the work of Shippen and May[8]. The model was scaled according to the subject's known weight and height. Segment sizes were estimated directly from the measured kinematic data, based on the relative positions of the joints.

The model received recorded kinematics in the form of joint rotation data, sampled at 240 Hz and resampled it to an internal rate of 120 Hz. These data are then filtered using a first-order Butterworth filter with a cut-off frequency of 20 Hz.No further pre-processing was performed.

During the inverse dynamical simulation, the model calculates the net torques and forces required at each joint to explain the measured kinematics, depending on the respective degrees of freedom. The individual muscle activations are then determined using the aforementioned Hill-type muscle model and an optimisation method that minimises overall muscle activation. Since only the activation of the arm muscles was considered, and not any muscular activation from the lower body, external ground reaction forces could be disregarded.

The muscle activation, muscle fibre length and muscle fibre velocity were extracted from the model, as well as the resampled kinematics. The model outputs were not filtered.

B Neural Network Models and Training

B.1 Neural Network Models

The InFoRM Network

The InFoRM network consists of a single reservoir of N neurons. Neurons are connected via the weights matrix $\boldsymbol{J}$, which has $p \cdot N$ non-zero entries taken from a Gaussian distribution with mean 0 and variance g^2/N. Here, p is the connection percentage and g is the scaling factor of the connection strength. All neurons receive output feedback of all $K = 27$ output signals, via weights $\boldsymbol{J}^{Fb}$ drawn from a uniform distribution between -1 and 1, and subsequently scaled by a factor γ_{in}. During training, the outputs $\hat{\boldsymbol{Y}}(t)$ are replaced by the target signals, effectively making them external inputs rather than feedback from the network. The look-ahead window $\boldsymbol{G_L}(t)$ provides goal information from the upcoming time steps, specifically from $t+1$ up to $t+L$, where $L = {}^{0.3}/_{\Delta t} = 36$, or up to the final time point T if $t+L > T$. This goal information is incorporated into the network through the goal weights $\boldsymbol{J}^G \in \mathbb{R}^{N\times L}$, drawn from a uniform distribution between -1 and 1.

The differential equation governing the neuron activations $\boldsymbol{x}$ thus reads:

$$\tau\dot{\boldsymbol{x}}(t) = -\boldsymbol{x}(t) + \boldsymbol{J}\phi\big(\boldsymbol{x}(t)\big) + \boldsymbol{J}^{Fb}\hat{\boldsymbol{Y}}(t) + \boldsymbol{J}^{G_L}\boldsymbol{G_L}(t), \tag{1}$$

where $\phi(\,\cdot\,)$ is the activation function, here realised by the hyperbolic tangent, and $\tau \sim \mathcal{N}(\mu_\tau,\ \mu_\tau\sigma_\tau)$ denotes the vector of time constants for each neuron, with μ_τ and σ_τ the hyperparameters specifying the mean, and the standard deviation as a fraction of μ_τ, respectively. This equation was solved numerically using Euler-forward integration to obtain the new neuron activations for each time step ($\Delta t = \frac{1}{120} = 0.0083$s). The activations were initialised randomly.

The network output $\hat{\boldsymbol{Y}}(t)$ is calculated from the neuron activations and a bias term according to:

$$\hat{\boldsymbol{Y}}(t) = \boldsymbol{W} \cdot \begin{bmatrix} \phi(\boldsymbol{x}(t)) \\ 1 \end{bmatrix}. \tag{2}$$

The output weights $\boldsymbol{W} \in \mathbb{R}^{(N+1)\times K}$ are the only weights that are updated during training (see below).

Control Network

In the standard control network, the inverse circuit is updated via the differential equation

$$\tau\dot{\boldsymbol{x}}^{inv}(t) = -\boldsymbol{x}^{inv}(t) + \boldsymbol{J}^{inv}\phi\left(\boldsymbol{x}^{inv}(t)\right) + \boldsymbol{J}^{G}\boldsymbol{G}(t) + \boldsymbol{J}^{G_L}\boldsymbol{G_L}(t) + \boldsymbol{J}^{Fb,inv}\hat{\boldsymbol{Y}}^{inv}(t) \tag{3}$$

The main difference to Equation (1) lies in the output $\hat{\boldsymbol{Y}}^{inv}(t)$, which only contains the $K^{inv} = 8$ efferent output signals. The goal information of the current time step is supplied separately via $\boldsymbol{G}(t)$, rather than via the output vector. The output of the inverse network $\hat{\boldsymbol{Y}}^{inv}(t)$ contains the $K^{inv} = 8$ efferent signals and is calculated in the same way as for the InFoRM network (see Equation (2)), substituting $\boldsymbol{Y}^{inv}$, $\boldsymbol{W}^{inv}$, and $\boldsymbol{x}^{inv}$ in the appropriate places.

The forward network gets updated according to:

$$\tau\dot{\boldsymbol{x}}^{fw}(t) = -\boldsymbol{x}^{fw}(t) + \boldsymbol{J}^{fw}\phi\left(\boldsymbol{x}^{fw}(t)\right) + \boldsymbol{J}^{In,fw}\hat{\boldsymbol{Y}}^{inv}(t) + \boldsymbol{J}^{Fb,fw}\hat{\boldsymbol{Y}}^{fw}(t). \tag{4}$$

Here, $\boldsymbol{J}^{In,fw}$ is the matrix of input weights for the signals coming from the inverse network, with values drawn from a uniform distribution between -1 and 1. The output of the forward network $\hat{\boldsymbol{Y}}^{fw}(t)$ contains the $K^{fw} = 16$ afferent signals and is calculated in the same way as for the InFoRM network (see Equation (2)), substituting $\boldsymbol{Y}^{fw}$, $\boldsymbol{W}^{fw}$, and $\boldsymbol{x}^{fw}$ in the appropriate places.

All matrices are initialised in the same way as for the InFoRM network. The output weights for both the inverse and forward network were updated during training.

Control+ Networks

To gradually bridge the gap between the Control and InFoRM architectures, we considered three modifications of the base Control network. In the Control+A network, the inverse network also receives the output feedback of the forward network to see if afferent feedback helps the network learn the muscle activation. This results in the following equation for the inverse network:

$$\tau\dot{\boldsymbol{x}}^{inv}(t) = -\boldsymbol{x}^{inv}(t) + \boldsymbol{J}^{inv}\phi\left(\boldsymbol{x}_j^{inv}(t)\right) + \boldsymbol{J}^{G}\boldsymbol{G}(t) + \boldsymbol{J}^{G_L}\boldsymbol{G_L}(t) + \boldsymbol{J}^{Fb,inv}\hat{\boldsymbol{Y}}^{inv}(t) + \boldsymbol{J}^{Fb2,inv}\hat{\boldsymbol{Y}}^{fw}(t) \tag{5}$$

Note that $\boldsymbol{J}^{Fb2,inv}$ is created in the same manner as $\boldsymbol{J}^{Fb,inv}$, but they are not numerically equal due to their random initialisation. The equation of the forward network remains unchanged (i.e. Equation (4)).

In the Control+G network, the equation of the inverse network is the same as that of the original control networks (Equation (3)), so the inverse network does not receive any afferent feedback. The forward network receives the goal information of the current time step $\boldsymbol{G}(t)$ as well as the lookahead window $\boldsymbol{G_L}(t)$, so the differential equation of the forward network becomes:

$$\tau\dot{\boldsymbol{x}}^{fw}(t) = -\boldsymbol{x}^{fw}(t) + \boldsymbol{J}^{fw}\phi\left(\boldsymbol{x}^{fw}(t)\right) + \boldsymbol{J}^{G}\boldsymbol{G}(t) + \boldsymbol{J}^{G_L}\boldsymbol{G_L}(t) + \boldsymbol{J}^{In,fw}\hat{\boldsymbol{Y}}^{inv}(t) + \boldsymbol{J}^{Fb,fw}\hat{\boldsymbol{Y}}^{fw}(t). \tag{6}$$

The Control+AG network incorporates both of these changes, such that both the inverse and forward networks receive all three signal types. The update equations for this network are therefore given by Equations (5) and (6).

B.2 Neural Network Training

During the training process, the networks receive the target output signals rather than the output feedback, enabling them to learn the output signals. To prevent overfitting and improve generalisation, additive noise $\boldsymbol{\xi}$ was introduced to the target signal $\boldsymbol{Y}$ during learning. The noise was defined as $\boldsymbol{\xi} \sim \mathscr{U}(-\eta\overline{|\boldsymbol{Y}|},\ \eta\overline{|\boldsymbol{Y}|})$, where $\mathscr{U}(\cdot)$ denotes a uniform distribution and η is a scalar hyperparameter controlling the noise amplitude.

Networks were trained using a recursive least squares algorithm[9]. This algorithm updates the weights after every time step. First, the output error $\boldsymbol{\varepsilon}$, a K-dimensional row vector, is calculated as the difference between the calculated output $\hat{\boldsymbol{Y}}(t)$ and the target output $\boldsymbol{Y}(t)$:

$$\boldsymbol{\varepsilon}(t) = \boldsymbol{Y}(t) - \hat{\boldsymbol{Y}}(t). \tag{7}$$

The output weights $\boldsymbol{W}(t)$ are initialised as zeroes and updated each time step based on the current neuron activation, the output error $\boldsymbol{\varepsilon}$, and the learning rate matrix $\boldsymbol{P}(t) \in \mathbb{R}^{N \times N}$. $\boldsymbol{P}(t)$ denotes an estimate of the inverse of the correlation matrix of $\boldsymbol{r}(t) = \begin{bmatrix} \phi(\boldsymbol{x}(t)) \\ 1 \end{bmatrix}$, with a regularisation term[9,10]:

$$\boldsymbol{W}(t+\Delta t) = \boldsymbol{W}(t) - \varepsilon(t) \frac{\boldsymbol{P}(t)\boldsymbol{r}(t)}{1+\boldsymbol{r}(t)^T \boldsymbol{P}(t)\boldsymbol{r}(t)}. \tag{8}$$

$\boldsymbol{P}(t)$ is updated according to:

$$\boldsymbol{P}(t+\Delta t) = \boldsymbol{P}(t) - \frac{\boldsymbol{P}(t)\boldsymbol{r}(t) \cdot (\boldsymbol{P}(t)\boldsymbol{r}(t))^T}{1+\boldsymbol{r}(t)^T \boldsymbol{P}(t)\boldsymbol{r}(t)}. \tag{9}$$

The networks were trained over n_{iter} iterations of each signal in the training set. The activations $\boldsymbol{x}$ of the neurons were initialised randomly at the start of each iteration. Each network was re-initialised and trained anew ten times, using a different random seed (81473, 90579, 12699, 91335, 63234, 9754, 27849, 54685, 95744, and 96481) each time.

The control models are trained in the same way, with the inverse and forward networks trained separately.

Hyperparameter tuning

Hyperparameters were optimised via Bayesian optimisation using the objective function

$$\mathscr{L} = \frac{1}{K} \sum_{k=1}^{K} \left(1 - \rho_k + \sqrt{\frac{1}{T} \sum_{t=1}^{T} \left(\hat{Y}_k(t) - Y_k(t) \right)^2} \right), \tag{10}$$

where ρ_k is the Pearson correlation coefficient between $\hat{Y}_k$ and Y_k over all time points. This function simultaneously optimises for temporal pattern alignment (via the correlation) and absolute error magnitude (via the root mean square error [RMSE]).

The optimisation was performed by training the networks on the training set (oscillations 1–6 of directions A, C, and E) and testing them on the validation set (oscillations 7–8 of directions A, C, and E). The search was repeated five times with different random seeds and with 250 iterations each. To avoid overfitting to random peaks in individual optimisation runs, we used the average of the five optimised hyperparameter sets to train the final model. An overview of the hyperparameters that were tuned can be found in Supplementary Table S3. Hyperparameter optimisation was performed separately for each subject and network type, using the same set of five random seeds (15762, 97059, 95715, 48537, and 80025) for all combinations.

References

1. Wagner, H. *et al.* Optimization Reduces Knee-Joint Forces During Walking and Squatting: Validating the Inverse Dynamics Approach for Full Body Movements on Instrumented Knee Prostheses. *Mot. Control.* **27**, 161–178, DOI: 10.1123/mc.2021-0110 (2023).
2. Kloock, L. *et al.* Joint contact forces during barefoot, minimal and conventional shod running are highly individual. *Sci. Reports* **15**, DOI: 10.1038/s41598-025-09174-w (2025).
3. Gerlach, M., De Graaf, M. L., Boström, K. J. & Wagner, H. Effect of advanced footwear technology spikes on lower limb kinetics and sprint performance. *Footwear Sci.* **17**, 69–77, DOI: 10.1080/19424280.2025.2458322 (2025).
4. Gerlach, M., De Graaf, M. L., Boström, K. J. & Wagner, H. Joint contact forces of the lower extremity during straight-line and curved sprinting. *Ger. J. Exerc. Sport Res.* DOI: 10.1007/s12662-024-01008-7 (2025).
5. De Graaf, M. L., Wagner, H., Mochizuki, L. & Le Mouel, C. Decreased spinal inhibition leads to undiversified locomotor patterns. *Biol. Cybern.* **119**, 12, DOI: 10.1007/s00422-025-01011-7 (2025).
6. Jäckle, K. *et al.* Revolutionizing clinical movement analysis: a novel approach using a wireless human motion capture system combined with computed myography in assessing coxarthrosis impact on joint contact forces. *Orthoplastic Surg.* **19**, 17–28, DOI: 10.1097/os9.0000000000000004 (2025).
7. Jäckle, K. *et al.* A novel and objective tool for determining total and shear joint contact forces after primary total hip arthroplasty. *J. Orthop.* **70**, 54–62, DOI: 10.1016/j.jor.2025.03.028 (2025).
8. Shippen, J. & May, B. A kinematic approach to calculating ground reaction forces in dance. *J. Dance Medicine & Sci.* **16**, 39 – 43, DOI: 10.1177/1089313X1201600106 (2012).

9. Haykin, S. S. *Adaptive Filter Theory*. Always Learning (Pearson, Upper Saddle River Boston Columbus San Francisco New York, 2014), fifth edition, international edition edn.

10. Sussillo, D. & Abbott, L. Generating Coherent Patterns of Activity from Chaotic Neural Networks. *Neuron* **63**, 544–557, DOI: 10.1016/j.neuron.2009.07.018 (2009).

Figures and Tables

Table S1. Number of times each muscle has been included as one of the eight main muscles used by a participant. The maximum is 12, as there are 12 participants.

Muscles	Counts
brachioradialis	12
infraspinatus	12
supraspinatus	12
biceps brachii caput breve	10
pectoralis major sternocostal	10
coracobrachialis	8
pronator teres humeral	7
latissimus dorsi first	6
teres minor	5
anconeus	4
pronator teres caput humeral	4
deltoideus clavicular	3
biceps brachii caput longum	2
latissimus dorsi	1

Table S2. Summary of the network schematics, optimised hyperparameters, and model performance across all network architectures. The schematics illustrate the test configurations of the unified InFoRM model, the standard Control model, and the Control+ variants. Large grey boxes denote the three neural network circuits: InFoRM, Inverse, and Forward. The yellow lines indicate the goal information ('G') represented by the desired movement kinematics, the red lines depict the efference ('Eff'), represented by the muscle activation, and the blue lines depict the afference ('Aff'), represented by the muscle length and velocity. The Control+ variants progressively incorporate additional information into the Control architecture. Specifically, Control+A provides additional sensory feedback to the inverse model (extra blue connection), Control+G supplies explicit goal information to the forward model (extra yellow connection), and Control+AG combines both modifications. These changes have been highlighted with the darker dashed lines. Below the schematics, the optimised number of neurons (N) and the number of training iterations (n_{iter}) are reported. Model performance is subsequently evaluated using the relative root mean square error (rRMSE) and the Pearson correlation coefficient (Corr.) for the Basic, Morphing, and Natural Transition test sets. Values are reported as estimated marginal means (EMM) with 95% confidence intervals across subjects. Green and red shading indicate the best and worst performance across architectures within each row, respectively.

		InFoRM		Control		Control+A		Control+G		Control+AG	

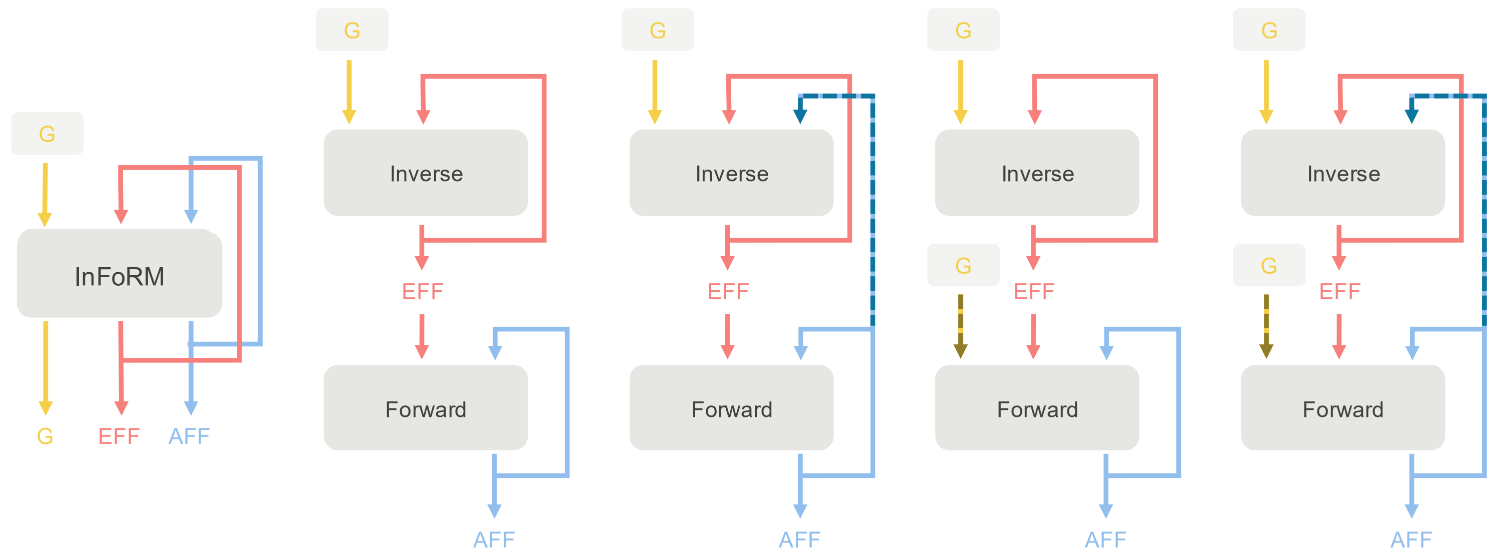

		EMM	95% CI	EMM	95% CI	EMM	95% CI	EMM	95% CI	EMM	95% CI
Nr. of Neurons (N)		465.47	[407.44 531.77]	544.15	[486.08 609.16]	570.08	[488.19 665.71]	582.49	[479.85 707.10]	647.54	[546.55 767.19]
Nr. of iterations (n_{iter})		3.50	[2.53 4.85]	11.68	[9.04 15.08]	14.66	[12.31 17.45]	10.97	[8.66 13.91]	10.33	[8.51 12.54]
Basic	rRMSE	.05	[.04 .05]	.12	[.11 .14]	.11	[.10 .12]	.07	[.06 .08]	.06	[.05 .07]
	Corr.	.96	[.95 .97]	.76	[.71 .80]	.83	[.79 .86]	.91	[.92 .94]	.93	[.92 .94]
Morphing	rRMSE	.12	[.11 .13]	.20	[.19 .22]	.18	[.17 .20]	.14	[.13 .15]	.14	[.13 .15]
	Corr.	.79	[.76 .82]	.54	[.49 .59]	.58	[.52 .62]	.72	[.68 .75]	.73	[.69 .76]
Natural Transitions	rRMSE	.14	[.12 .15]	.29	[.26 .32]	.22	[.20 .25]	.19	[.17 .21]	.18	[.16 .20]
	Corr.	.78	[.75 .81]	.45	[.39 .51]	.53	[.47 .58]	.67	[.62 .71]	.69	[.65 .73]

Table S3. List of hyperparameters that have been optimised for the InFoRM and Control models.

Parameter Name		Description
**InFoRM**	**Control**	
N	N^{inv}, N^{fw}	Number of neurons in the reservoir
g	g^{inv}, g^{fw}	Scaling factor for connection strength (spectral radius)
p	p^{inv}, p^{fw}	Proportion of connected neurons in the reservoir
μ_τ	μ_τ^{inv}, μ_τ^{fw}	Mean time constant for neuron dynamics
σ_τ	σ_τ^{inv}, σ_τ^{fw}	Standard deviation of time constant, as a fraction of μ_τ
n_{iter}	n_{iter}	Number of training iterations
α	α	Learning rate for weight updates
δ	δ	Regularisation parameter
η	η	Noise scaling of the input/output feedback factor during training
γ_{in}	γ_{in}	Factor scaling the input/output feedback weights

Table S4. Post hoc comparisons for the effect of network architecture on the relative root-mean square error (rRMSE) and the correlation between the InFoRM network and all Control networks for each of the three test sets.

Comparison		rRMSE					Correlation				
Network 1	Network 2	DF1	DF2	F	p	η_p^2	DF1	DF2	F	p	η_p^2
Basic											
InFoRM	Control	1	537	1005.8	$< .001$	0.65	1	580	1112.2	$< .001$	0.66
InFoRM	Control+A	1	537	793.49	$< .001$	0.60	1	580	705.85	$< .001$	0.55
InFoRM	Control+G	1	537	199.47	$< .001$	0.27	1	580	219.11	$< .001$	0.27
InFoRM	Control+AG	1	537	72.313	$< .001$	0.12	1	580	82.021	$< .001$	0.12
Control	Control+A	1	537	24.617	$< .001$	0.04	1	580	46.398	$< .001$	0.07
Control	Control+G	1	537	310.18	$< .001$	0.37	1	580	333.76	$< .001$	0.37
Control	Control+AG	1	537	525.78	$< .001$	0.49	1	580	566.94	$< .001$	0.49
Control+A	Control+G	1	537	179.38	$< .001$	0.25	1	580	132.97	$< .001$	0.19
Control+A	Control+AG	1	537	358.82	$< .001$	0.4	1	580	293.05	$< .001$	0.34
Control+G	Control+AG	1	537	29.777	$< .001$	0.05	1	580	31.439	$< .001$	0.05
Morphing											
InFoRM	Control	1	556	551.94	$< .001$	0.50	1	583	788.32	$< .001$	0.57
InFoRM	Control+A	1	556	383.8	$< .001$	0.41	1	583	636.2	$< .001$	0.52
InFoRM	Control+G	1	556	85.691	$< .001$	0.13	1	583	101.75	$< .001$	0.15
InFoRM	Control+AG	1	556	77.669	$< .001$	0.12	1	583	77.502	$< .001$	0.12
Control	Control+A	1	556	20.147	$< .001$	0.03	1	583	8.3687	$< .01$	0.01
Control	Control+G	1	556	214.33	$< .001$	0.28	1	583	326.03	$< .001$	0.36
Control	Control+AG	1	556	224.15	$< .001$	0.29	1	583	374.39	$< .001$	0.39
Control+A	Control+G	1	556	108.98	$< .001$	0.16	1	583	230.5	$< .001$	0.28
Control+A	Control+AG	1	556	116.7	$< .001$	0.17	1	583	271.45	$< .001$	0.32
Control+G	Control+AG	1	556	0.1666	.683	0	1	583	1.6646	.198	0
Natural Transitions											
InFoRM	Control	1	590	1538	$< .001$	0.72	1	566	836.47	$< .001$	0.60
InFoRM	Control+A	1	590	1045.5	$< .001$	0.64	1	566	330.39	$< .001$	0.37
InFoRM	Control+G	1	590	285.01	$< .001$	0.33	1	566	169.37	$< .001$	0.23
InFoRM	Control+AG	1	590	184.35	$< .001$	0.24	1	566	120.97	$< .001$	0.18
Control	Control+A	1	590	47.199	$< .001$	0.07	1	566	106.74	$< .001$	0.16
Control	Control+G	1	590	489.04	$< .001$	0.45	1	566	253.22	$< .001$	0.31
Control	Control+AG	1	590	658.87	$< .001$	0.53	1	566	331.98	$< .001$	0.37
Control+A	Control+G	1	590	233.39	$< .001$	0.28	1	566	28.547	$< .001$	0.05
Control+A	Control+AG	1	590	352.82	$< .001$	0.37	1	566	56.12	$< .001$	0.09
Control+G	Control+AG	1	590	11.517	$< .001$	0.02	1	566	4.5422	$< .05$	0.01

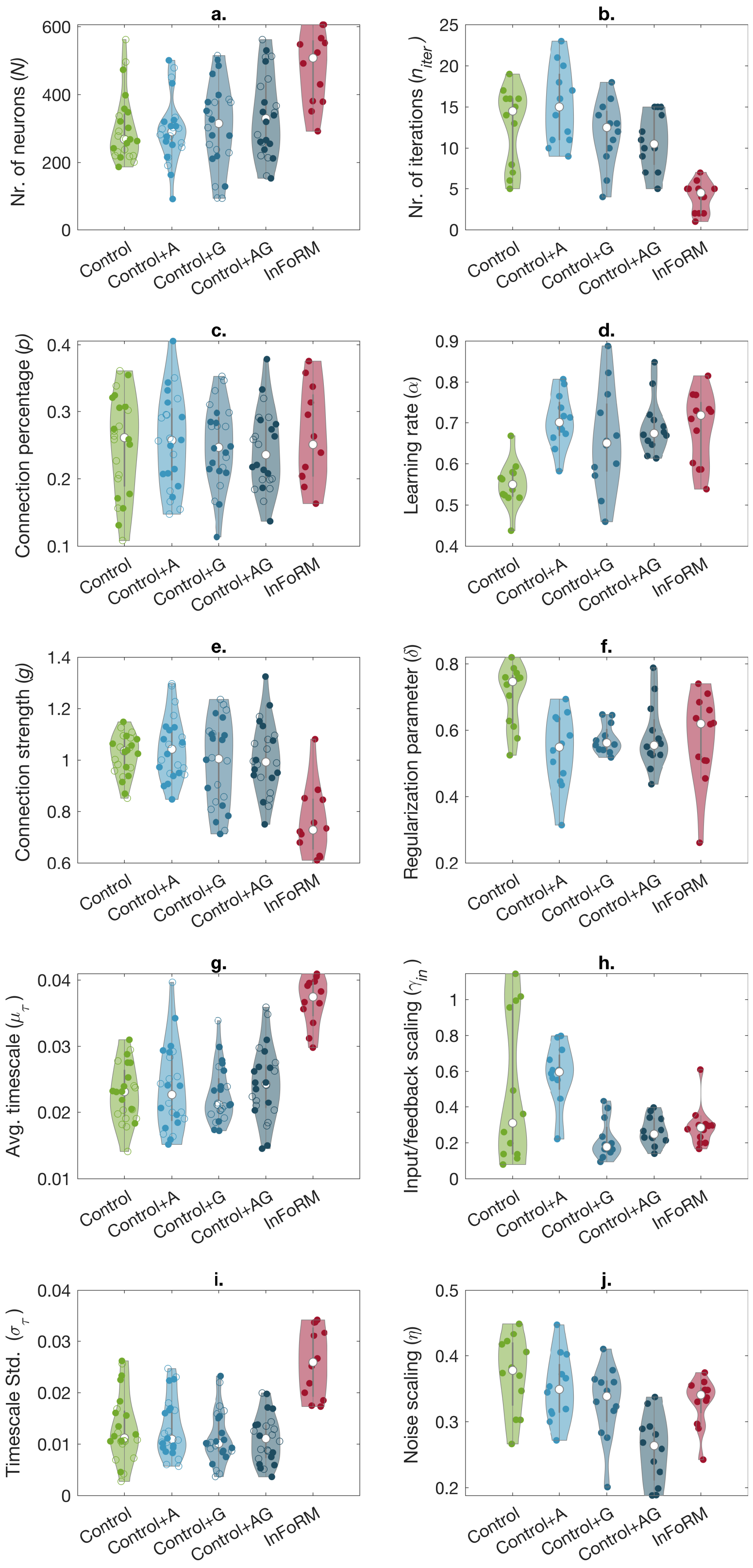

Figure S1. The optimised hyperparameters for all network architectures. Each circle represents one subject. For the Control networks, the hyperparameters are shown for the inverse (filled circles) and forward (open circles) models separately when applicable. The violin outline is created over the combined set of forward and inverse function values.

Table S5. Post hoc comparisons between the InFoRM network and all Control networks for the number of neurons and the number of training iterations. NB: None of the differences between the various control architectures (not shown) were significant.

Network	EMM	95% CI	DF1	DF2	F	p	η_p^2
Number of neurons (N)							
InFoRM	465.47	[407.44, 531.77]					
Control	511.15	[486.08, 609.16]	1	54	3.3659	0.43	0.06
Control+A	570.08	[488.19, 665.71]	1	54	4.9422	0.27	.08
Control+G	582.49	[479.85, 707.10]	1	54	3.8979	0.39	.07
Control+AG	647.54	[546.55, 767.19]	1	54	8.0262	0.06	.13
Number of training iterations (n_{iter})							
InFoRM	3.50	[2.53, 4.85]					
Control	11.68	[9.04, 15.08]	1	55	45.517	$< .001$	.453
Control+A	14.66	[12.31, 17.45]	1	55	79.482	$< .001$	.591
Control+G	10.97	[8.66, 13.91]	1	55	34.238	$< .001$	.384
Control+AG	10.33	[8.51, 12.54]	1	55	32.056	$< .001$	.368
